# Prediction and optimization of stretch flangeability of advanced high strength steels utilizing machine learning approaches

**DOI:** 10.1038/s41598-025-00786-w

**Published:** 2025-05-10

**Authors:** Tianyang Li, Zheng Yang, Junyi Cui, Wenjie Chen, Rami Almatani, Yingjie Wu

**Affiliations:** 1 Sichuan University - Pittsburgh Institute (SCUPI), Sichuan University, Chengdu, 610207 China; 2 Department of Materials Science and Engineering, National University of Singapore, Singapore, 117575 Singapore; 3 Advanced Materials Technologies Institute, King Abdulaziz City for Science and Technology, Riyadh, 11442 Saudi Arabia

**Keywords:** Stretch-flangeability, Advanced high strength steels, Composition-structure-property relationship, Machine learning, Multiple objective optimization, Computational methods, Mechanical properties

## Abstract

Advanced high strength steels (AHSS) exhibit diverse mechanical properties due to their complex chemical compositions and microstructures. Existing machine learning (ML) studies often focus on specific steel grades, limiting generalizability in predicting and optimizing AHSS properties. Here, an ML framework was presented to predict and optimize the stretch-flangeability of AHSS based on composition-microstructure-property correlations, using datasets from 212 steel conditions. Support vector machine, symbolic regression, and extreme gradient boosting models accurately predicted hole expansion ratio (HER), ultimate tensile strength (UTS), and total elongation (TE). Shapley additive explanations revealed the importance of bainite volume fraction (VB), carbon content (C), and chromium content (Cr) for HER, UTS, and TE, respectively. Multi-objective optimization generated 252 optimized conditions with improved comprehensive mechanical properties. The best optimized chemical compositions (0.12wt.% C-1.10Mn-0.15Si-0.47Cr) along with the carbon equivalent (CE) of 0.44 wt.%, and microstructural features (7.2% ferrite, 44.5% bainite, 40.5% martensite, and 7.8% tempered martensite) yielded HER of 119.8%, UTS of 1013.5 MPa, and TE of 22.7%. This systematic framework enables efficient prediction and optimization of material properties (especially HER), with potential applications across various fields of materials science.

**Supplementary Information:**

The online version contains supplementary material available at 10.1038/s41598-025-00786-w.

## Introduction

Advanced high strength steels (AHSS) have gained a widespread application in the automotive industry due to their superior performances in fuel efficiency enhancement, green-house gas emission reduction, crashworthiness improvement, and vehicle weight reduction^1^. Dual phase (DP) steels, a typical representative of AHSS, are characterized by a soft ferrite matrix with dispersed martensite islands, achieved through carefully selected compositions and strictly controlled processing^2^. These microstructural features contribute to an optimal balance between strength and ductility. The mechanical properties of DP steels include low yield strength (YS), high ultimate tensile strength (UTS), low yield to tensile strength ratio (YS/UTS), continuous yielding, and high initial work hardening ratio^3^. Complex phase (CP) steels, as the potential alternative to DP steels, are identified by a ferrite-bainite matrix with a limited amount of martensite^4^, tempered martensite^1,5,6^, cementite^7^or retained austenite^8^. Regarding mechanical properties, CP steels possess more uniform strain hardening behavior^9^, superior fatigue resistance^10^, and better energy absorption capacity^10,11^ compared to DP steels.

Stretch-flangeability is a crucial parameter in the fabrication of complex-shaped automotive parts, such as door panels, fenders, and body-in-white components, requiring extensive edge stretching during forming operations^12^. It is evaluated by hole expansion ratio (HER), a significant parameter in assessing the local ductility of DP or CP steels, especially during the complex shape forming process in the automotive industry^12,13^. HER values are determined by conducting hole expansion tests (HET) following ISO 16630^14^. During the test, the pre-punched central hole is expanded by a 60° conical punch and the test is stopped until the first through-thickness edge crack is observed on the hole surface. HER results are given by Eq. (1)^14^,

1 $${\text{HER}}\;{\text{ = }}\;\frac{{{{\text{D}}_{\text{f}}}\;{\text{ - }}\;{{\text{D}}_{\text{o}}}}}{{{{\text{D}}_{\text{o}}}}}\; \times \;{\text{100}}\%$$

where, D_o_ and D_f_ represent the initial and final hole diameters before and after HET, respectively. Higher HER values indicate the greater sheared-edge ductility, which are optimum features for DP or CP steels in applications such as stretch flanging and deep drawing.

Earlier literature focused on key factors governing the stretch-flangeability of AHSS. Paul^13^ summarized that HER is influenced by microstructure features, mechanical properties, hole preparation approaches and punch geometries. For instance, in order to precisely predict HER values without performing HETs, linear or nonlinear correlations were established based on simple uniaxial tensile properties, including normal anisotropy ($\:\overline{\text{R}}$)^15,16^, post uniform elongation (PUE)^15,17^, strain hardening ratio (n)^18^, and UTS^15,17^. Also, some other mechanical property, such as initial fracture energy, was found to be closely related to stretch-flangeability^12^. However, these relationships are only valid for several AHSS grades, such as interstitial free (IF), bake hardened (BH), mild, and high strength low alloy (HSLA) steels, but not for DP or CP steels. Since the complex microstructures of AHSS, often characterized by multiple phases with different resulting mechanical properties, make it difficult to predict their stretch-flangeability using traditional empirical models. These models often fail to attain the complicated relationships between microstructure features and mechanical behavior, leading to inaccurate predictions and limited applicability across different AHSS grades^19^.

To address this challenge, researchers have been studying the applications of machine learning (ML) techniques to develop predictive models for mechanical properties based on microstructure-property correlations. ML algorithms, such as regression, neural networks, and ensemble methods, have shown great potential in materials science for their ability to learn complex, non-linear relationships from large datasets^20–22^. These algorithms can effectively reveal the correlations between microstructural features and mechanical properties, enabling the development of accurate and generalizable predictive models^22,23^.

Several studies have successfully applied ML techniques to predict different mechanical properties of metal and alloys. For example, Lee et al.^24^ predicted the UTS and TE of medium Mn steels, such as a Fe-5.5Mn-0.2 C-0.3Si steel, applying a boosted decision tree regression model. Bhandari et al.^25^ developed a random forest regression model to predict the high-temperature YS of MoNbTaTiW and HfMoNbTaTiZr high entropy alloys. Ma et al.^26^ used a random forest algorithm to predict the room-temperature fracture toughness of Nb-Si based alloys based on their chemical compositions. Bao et al.^27^ employed a support vector regression model to predict the high cycle fatigue life of addictively manufactured Ti-6 Al-4 V alloys based on their defect characteristics. Moreover, Wang et al.^28^ applied various ML approaches to predict the creep rupture life of Cr-Mo steels. In the investigation, they developed a random forest model which demonstrated the highly precise prediction of Manson-Succop Parameter (MSP).

However, the application of ML to predict the stretch-flangeability of AHSS remains largely unexplored. The limited studies available on predicting stretch-flangeability using ML have primarily focused on several specific AHSS grades. For instance, Lee et al.^29^ integrated a generative adversarial imputation network with an extra tree regressor model to predict HER results of AHSS based on their experimental tensile properties. However, this study did not consider the microstructural features, which may play a crucial role in determining stretch-flangeability. Li et al.^30^ tried to evaluate the hole expansion performances of AHSS via different ML approaches according to their alloying elements and experimental microstructure features. While this study demonstrated the potential of ML to predict stretch-flangeability based on microstructure data, it was limited to several AHSS grades.

In this study, 212 datasets from earlier studies^15,17,31–42,12,43–61^ containing chemical compositions, microstructural characteristics and mechanical properties of AHSS, especially DP and CP steels, were analyzed and processed, completing missing values via multivariate imputation by chained equations (MICE). Then three ML algorithms, such as support vector machine (SVM), symbolic regression (SR) and extreme gradient boosting (XGBoost) were employed to determine correlations between HER, UTS or TE with compositions, including carbon (C), carbon equivalent (CE), manganese (Mn), silicon (Si), and chromium (Cr), and volume fractions of different phases, such as ferrite (VF), bainite (VB), martensite (VM) and tempered martensite (VTM). Here, CE is a measure that quantifies the combined effect of various alloying elements on the hardenability of AHSS, which is assumed to influence HER in this study. CE can be expressed by Eq. (2)^62^.

2 $$\:\text{CE}\text{}\text{=}\text{}\text{C}\text{}\text{+}\text{}\frac{\text{Mn}}{\text{6}}\text{}\text{+}\text{}\frac{\text{Cr}\text{}\text{+}\text{}\text{Mo}\text{}\text{+}\text{}\text{V}}{\text{5}}\text{}\text{+}\text{}\frac{\text{Ni}\text{}\text{+}\text{}\text{Cu}}{\text{15}}$$

where, C, Mn, Cr, Mo, V, Ni, and Cu represent the weight percentages of carbon, manganese, chromium, molybdenum, vanadium, nickel, and copper, respectively. Further, a multiple objective optimization (MOO) approach was utilized to design AHSS with an optimal combination of HER, UTS and TE. This method allows for the appropriate volume percentages of multiple phases. By leveraging the power of ML algorithms and MOO techniques, this study aims to provide valuable insights into the complex composition-structure-property relationships in AHSS and guide the development of new AHSS grades with improved performance.

## Results

**Fig. 1:**
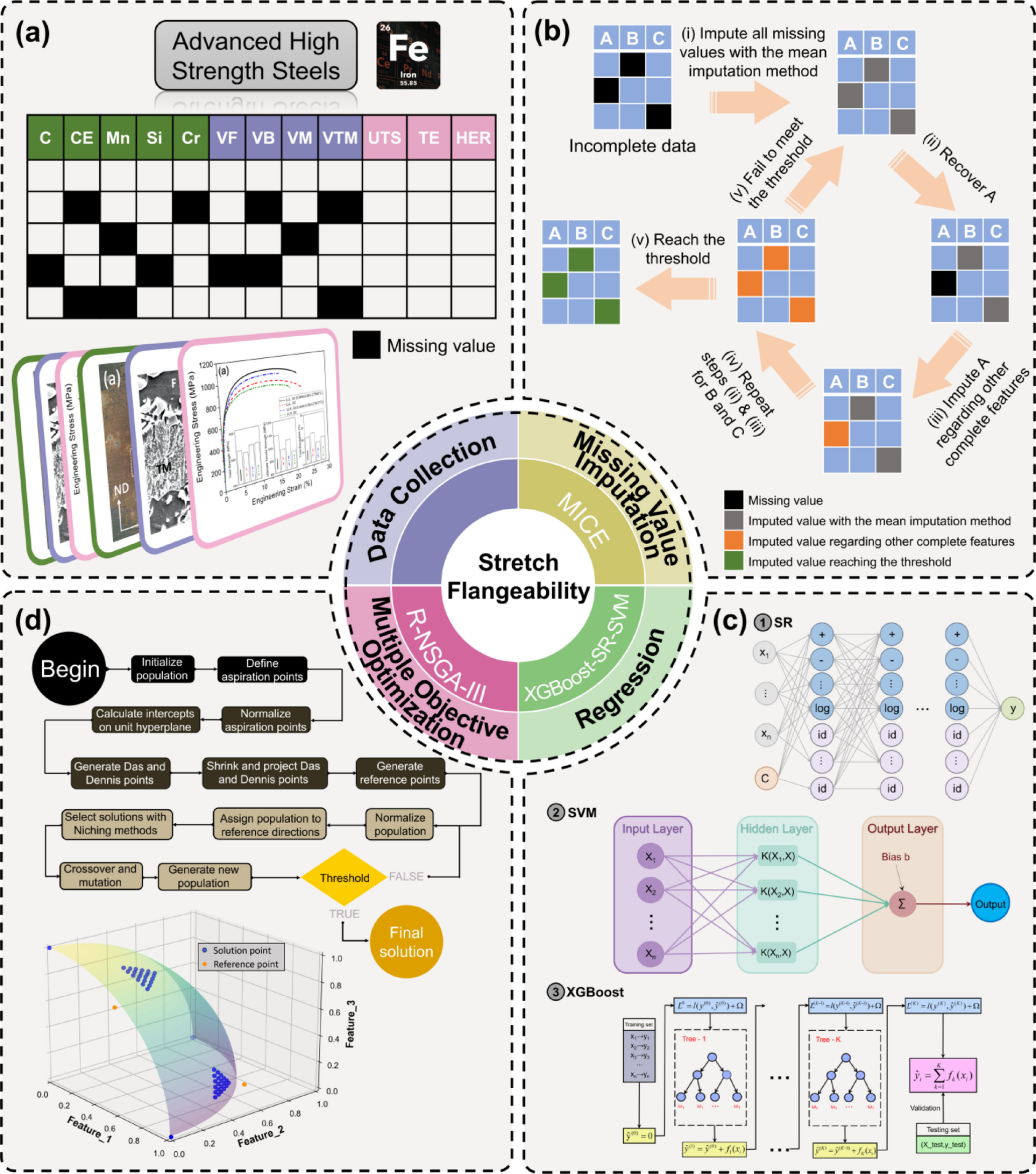
Overview of the prediction and optimization framework for the stretch-flangeability of AHSS. **(a)** Data collection: gathering chemical compositions, microstructural characteristics and mechanical properties from 212 steel conditions. (**b)** Missing value imputation: employing MICE methods to address incomplete datasets, with missing value analysis and cross-validation for method selection. (**c)** Regression: utilizing an interpretable ML framework utilizing SVM, SR, or XGBoost algorithm, coupled with SHAP for feature importance interpretation. (**d)** MOO: implementing R-NSGA-III to simultaneously optimize HER, UTS, and TE while considering compositional and microstructural constraints.

The proposed prediction and optimization framework for the stretch-flangeability of AHSS is illustrated in Fig. 1. A comprehensive four-step approach was implemented, including data collection (Fig. 1a), missing value imputation (Fig. 1b), regression (Fig. 1c), and MOO (Fig. 1d). In the data collection phase (Fig. 1a), 212 steel conditions were investigated, forming datasets containing 29 features including chemical composition content in weight% (iron (Fe), niobium (Nb), Ni, phosphorus (P), nitrogen (N), boron (B), sulfur (S), V, titanium (Ti), Cu, molybdenum (Mo), aluminum (Al), C, CE, Mn, Si, and Cr), microstructure characteristics (ferrite grain size (d_F_), VF, VB, VM and VTM) and mechanical properties (HER, YS, UTS, UE, PUE, TE, and reduction in area (RA)). Only 28 datasets were complete, highlighting the necessity of missing value analysis and imputation. For missing value imputation (Fig. 1b), MICE was employed. Three MICE methods (multiple linear regression (MLR), Lasso, and Ridge regression) were utilized and evaluated through cross-validation tests. The regression analysis step (Fig. 1c) employed three advanced ML algorithms, SVM with radial basis function kernel, SR using multigene genetic programming, and XGBoost employing gradient boosting decision tree. These methods were chosen for their ability to determine complex, non-linear relationships between chemical compositions and microstructural features with mechanical properties. To enhance interpretability, shapley additive explanations (SHAP) was utilized to explain the impact and importance of each input feature. Finally, a MOO approach (Fig. 1d) was implemented using the reference point based non-dominated sorting genetic algorithm III (R-NSGA-III) to simultaneously optimize HER, UTS, and TE while considering relevant constraints on both chemical compositions and microstructural phase fractions.

### Performance evaluation for MICE

To better illustrate the performance of different imputation approaches in MICE, a 10-fold cross-validation analysis was conducted in this study. Table 1compares the Q^2^ values for each imputed variable with MLR, Lasso and Ridge. The Ridge regression in MICE demonstrated the best performance in predicting missing values.

**Fig. 2:**
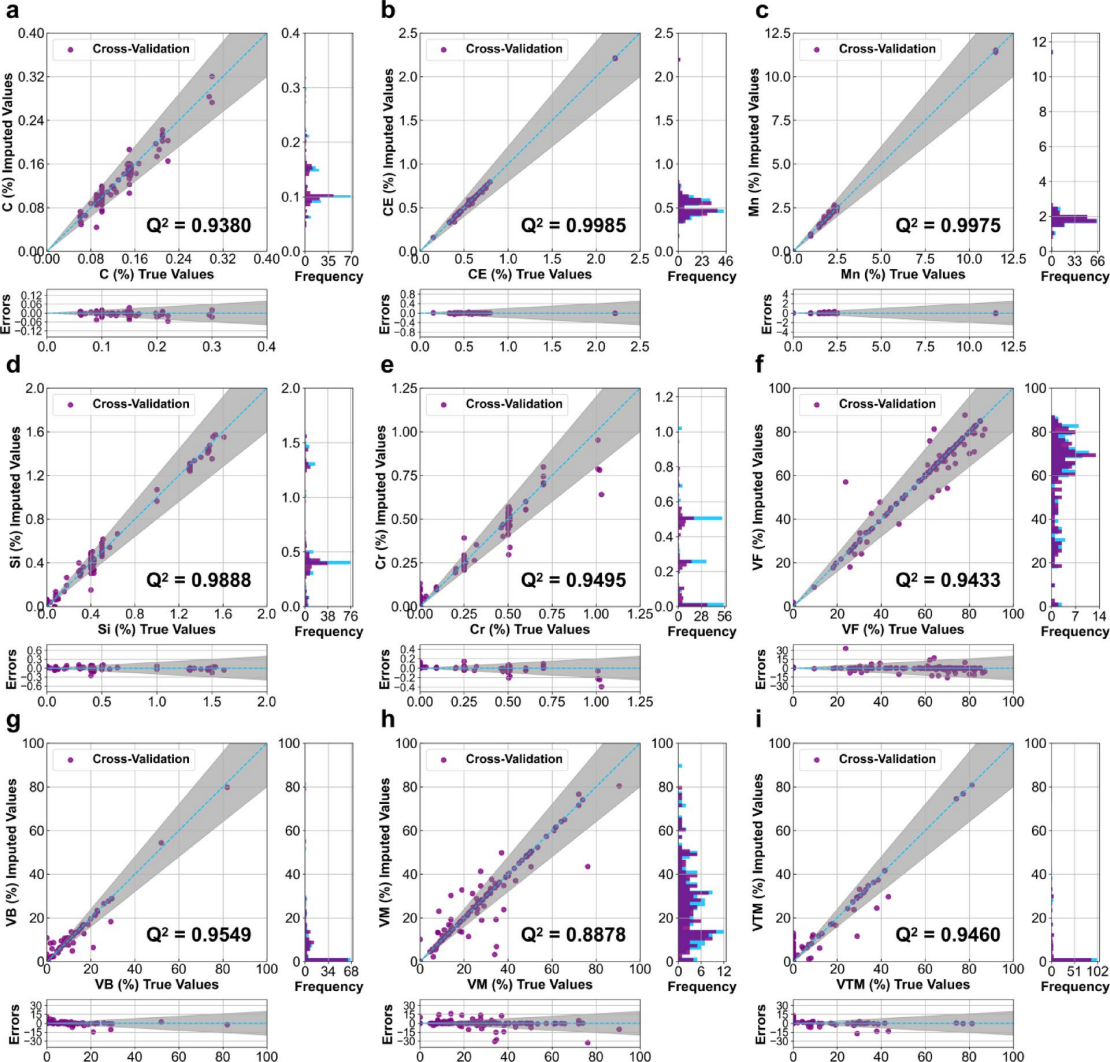
Evaluation of Ridge method in MICE for compositions and microstructure features. **(a-i)** Detailed comparison and error scatter plots, as well as frequency histogram plots illustrating the relationships between imputed values (in purple) and true values (in blue) for individual microstructural characteristics using the Ridge regression method in MICE: (**a)** C, (**b)** CE, (**c)** Mn, (**d)** Si, (**e)** Cr, (**f)** VF, (**g)** VB, (**h)** VM, (**i)** VTM. Note: The shaded regions represent an error range of ± 20%.

Figures 2a-i further illustrates the effectiveness of the Ridge method. Significantly, the 10-fold cross-validation Q^2^ values for C, CE, Mn, Si, Cr, VF, VB, VM, and VTM were 0.9380, 0.9985, 0.9975, 0.9888, 0.9495, 0.9433, 0.9549, 0.8878, and 0.9460 (Table 1), respectively, with an average of 0.9560. These results indicate that the Ridge regression in MICE can predict unknown values with the superior accuracy across all compositions and microstructure features. Also, the values of frequency for C, CE, Mn, Si, Cr, VB and VTM mainly concentrate around one or few values (Fig. 2a-e, g and i), while those of VF and VM are more diverse (Fig. 2f and h), with VF values having more larger values than VM. According to the shaded regions (points with errors within ± 20%), VM seem to have comparatively more deviation from the real values, which is consistent with higher Q^2^ values compared to those of others. Overall, the scatter figures show that all imputed values not only fit the features well, but also have small errors with the real values.

**Table 1:** Comparisons of evaluation of three regression models (MLR, Lasso and Ridge) in MICE for both chemical composition contents and microstructural characteristics.

	C	CE	Mn	Si	Cr	VF	VB	VM	VTM
MLR	0.9494	0.9952	0.9864	0.9759	0.7392	0.9094	0.8292	0.8728	0.9318
Lasso	−0.0126	0.5711	0.9379	0.1450	−0.0566	0.9586	0.9238	0.9186	0.9418
Ridge	0.9380	0.9985	0.9975	0.9888	0.9495	0.9433	0.9549	0.8878	0.9460

### Hyperparameter optimization and training process

Three regression methods, including SVM, SR and XGBoost were utilized to investigate the relationships between compositions (C, CE, Mn, Si, and Cr) and microstructural features (VF, VB, VM, and VTM) with mechanical properties (HER, UTS and TE) of AHSS. To optimize the performance of the SVM and XGBoost models, hyperparameter tuning was performed using Bayesian optimization, which aimed to maximize the 10-fold cross validation Q^2^ values. While to acquire the best SR models, we generated a large number of individuals in the SR population and select the ones possessing the best predicting performance, namely, largest Q^2^ in the 10% validation set.

For SVM, regularization parameter (C), margin of tolerance (epsilon) and kernel coefficient (gamma) were optimized, while in term of SR, the best symbolic models were tuned with their performances of in validation set. Regarding XGBoost, the number of decision trees (n_estimators), maximum depth of each tree (max_depth), samples’ ratio (subsample), inputs’ ratio (colsample_bytree), and step size at each iteration (learning_rate) were optimized. Additionally, based on prior experience and background information, the kernel function type (kernel) in SVM was set to ‘rbf’. The population size (pop_size), number of generations (num_gen) and mathematical operators (functions.name) in SR were set to 500, 800 and {‘times’, ‘minus’, ‘plus’, ‘neg’, ‘exp’, ‘negexp’, ‘gauss’, ‘sin’, ‘cos’, ‘tan’, ‘sinh’, ‘cosh’, ‘tanh’}, and the base estimator (booster) in XGBoost was set to ‘gbtree’. These adjustments to the hyperparameters were intended to strike a balance between the fitting performance and prediction robustness of the SVM, SR, and XGBoost models.

It is important to note that, even with identical optimized parameters, the results generated by SR using GPTIPS2 were still uncertain. Consequently, the SR results presented for the complete set were the best ones from multiple trials, while the SR results for 10-fold cross-validation were randomly collected.

### Prediction of mechanical properties through ML approaches

**Fig. 3:**
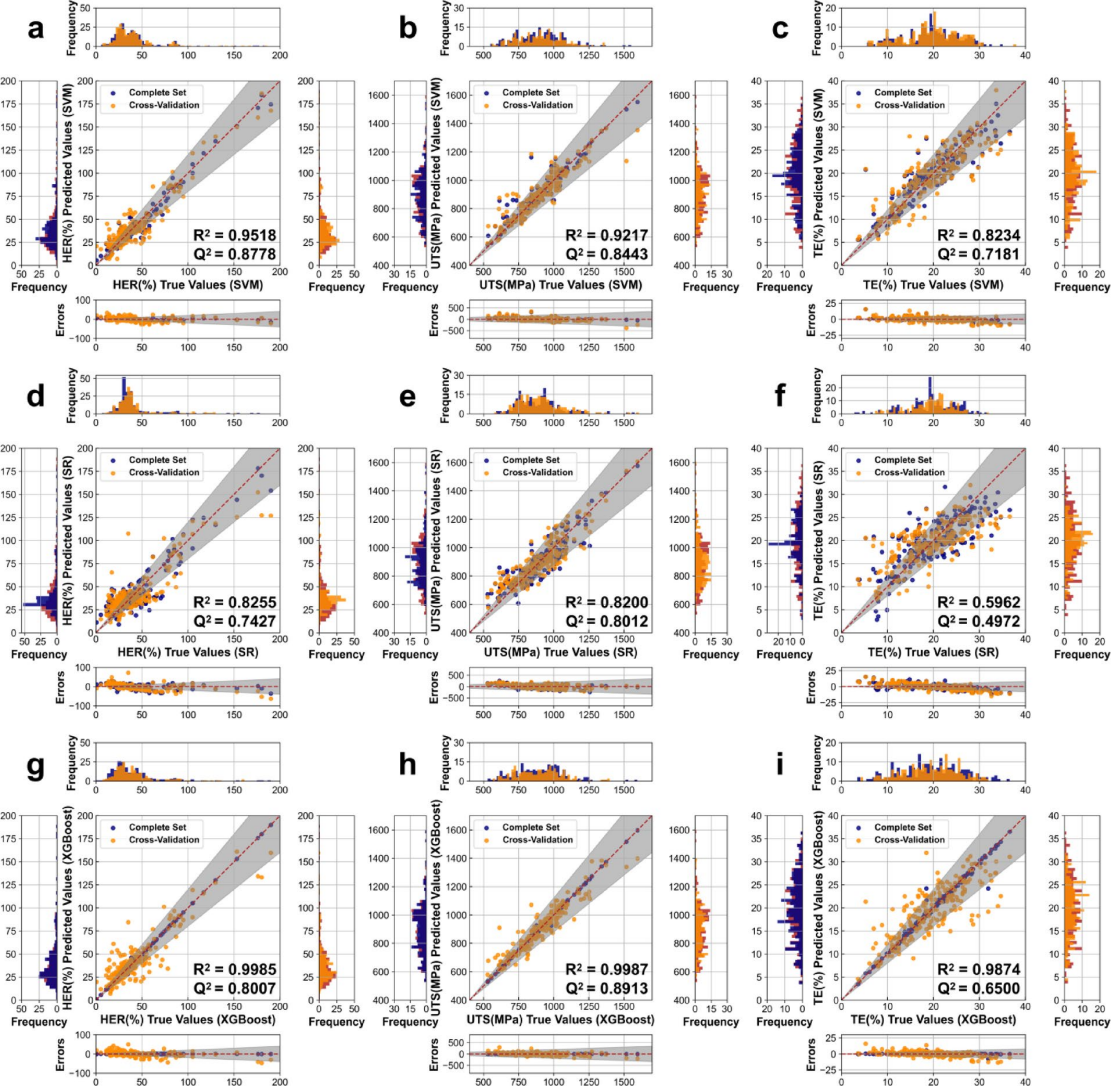
Performance evaluation of ML models for predicting key mechanical properties of AHSS. **(a-c)** SVM model performance: (**a)** HER, (**b)** UTS, and (**c)** TE. (**d-f)** SR model performance: **(d)** HER, (**e)** UTS, and (**f)** TE. (**g-i)** XGBoost model performance: (**g)** HER, (**h)** UTS, and (**i)** TE. There exist detailed comparison and error scatter plots, as well as frequency histogram plots to illustrate the relationships between true values (in red), predicted values in complete sets (in dark blue) and cross-validation sets (in dark orange). Note: The shaded regions represent an error range of ± 20%.

Figure 3 illustrates the estimating performances of SVM, SR, and XGBoost models for HER, UTS and TE predictions of AHSS. Concerning HER predictions (Fig. 3a, d and g), scatter plots show small deviations from the true values for both the complete set and cross-validation. The frequency distribution plots and shaded regions (representing an error range of ± 20%) indicate that HER predictions from SR and XGBoost are similarly distributed around the shaded regions, although SR’s estimations from the complete set and cross-validation appear to be more coherent. SVM’s estimated scatters are more concentrated around the real values, and the results from the complete set and cross-validation are similar, demonstrating its accurate and robust estimation. Values of R^2^ for HER predictions are generally larger than 0.82, indicating that the employed ML models can fit the HER values with high accuracy. Moreover, the 10-fold cross-validation Q^2^ values for all models are larger than 0.74, suggesting good predictive abilities. The XGBoost model demonstrated superior fitting performance on the training set with an R^2^ of 0.9985, significantly outperforming both SVM and SR models having R^2^ values of 0.9518 and 0.8255, respectively. However, the cross-validation Q^2^ values for XGBoost and SR are only 0.8007 and 0.7427, respectively, showing relatively high predictive ability for unknown data. Notably, SVM regression for HER yields a Q^2^ of 0.8778, which is not only close to its R^2^ value, but also prominently higher than those of SR and XGBoost. These metrics indicate that SVM provides the best estimations with minimal overfitting bias, and the close alignment between R^2^ and Q^2^ underscores its potential for generalization.

For UTS predictions (Fig. 3b, e and h), the scatter distributions show excellent coherence within the shaded regions (errors within ± 20%) for all three ML models, and the distinctions between the complete set and cross-validation are small, indicating good estimating ability. XGBoost’s estimations of UTS are particularly close to the real values. The R^2^ values for UTS predictions typically exceed 0.82, suggesting that the ML models provide constructive estimations. The 10-fold cross-validation Q^2^ values are approximately 0.80, demonstrating reliable predictive capability across different datasets. As exhibited in Fig. 3, the R^2^ values for SVM and SR are 0.9217 and 0.8200, respectively, while the Q^2^ values are 0.8443 and 0.8012, respectively. Therefore, the estimating accuracy of SVM and SR is similar in cross-validation conditions, with SVM showing an advantage in the complete set and SR superior in less distinction between R^2^ and Q^2^. XGBoost’s estimating performance is notably leading in both the complete set and cross-validation, with R^2^ and Q^2^ values of 0.9987 and 0.8913, respectively, illustrating the highest regression accuracy and the best ability of predicting unobserved data.

In terms of TE predictions, the scatter plots in Fig. 3c, f and i show that SVM, SR, and XGBoost all have multiple cross-validation points outside the shaded regions (errors within ± 20%), indicating that the predicting performances for TE are not as satisfactory as those for HER and UTS. The scatter and residual plots for the XGBoost model show prominent distinctions between the complete set and cross-validation, which may be related to overfitting issues. SR exhibits the lowest R^2^ (0.5962) and Q^2^ (0.4972) values among three ML models for TE predictions. The R^2^ value indicates that SR cannot fit TE with good accuracy, while the small difference between R^2^ and Q^2^ suggests SR does not overfit. The R^2^ values for TE predictions using SVM and XGBoost are 0.8234 and 0.9874, respectively, which are greater than 0.82, and their Q^2^ values are 0.7181 and 0.6500, respectively, which are no lower than 0.65. These metrics suggest that SVM and XGBoost can provide more acceptable information on TE. For SVM, though its R^2^ value is slightly lower than that of XGBoost, its larger Q^2^ value and less distinction between R^2^ and Q^2^ indicates that SVM might have a less severe overfitting problem, which is exactly needed for predicting and optimizing AHSS’s properties.

In summary, SVM demonstrated the best performance for HER predictions, with a balanced R^2^ (0.9518) and Q^2^ (0.8778) values, indicating its ability to both fit the data well and predict unseen data samples with the highest accuracy. XGBoost excelled in UTS predictions, with an R^2^ of 0.9987 and a Q^2^ of 0.8913, illustrating the highest regression accuracy and the least amount of overfitting. For TE predictions, all models had lower R^2^ and Q^2^ values compared to HER and UTS, while SVM, with an R^2^ of 0.8234 and a Q^2^ of 0.7181, takes an advantage over others in predicting unknown values. These findings highlight the importance of selecting appropriate ML algorithms for predicting different mechanical properties and the need for careful evaluation of model performance using both R^2^ and Q^2^ metrics to assess fitting accuracy and generalization ability. As a result, the ML models with the best predictive performances for HER, UTS and TE, namely, SVM models for HER and TE, as well as XGBoost model for UTS, are applied in the following process of MOO to provide the most reliable virtual samples.

In addition, SR models offer the advantage of generating interpretable mathematical expressions that describe the relationships between compositions (C, CE, Mn, Si, and Cr) and microstructural features (VF, VB, VM and VTM) and mechanical properties (HER, UTS and TE). The SR-derived equations are given in Eqs. (3)-(5),

3 $$\begin{aligned}\text{H}\text{E}\text{R}&={\text{e}}^{-\text{C}\text{r}-\text{s}\text{i}\text{n}\left(\text{S}\text{i}\right)}\cdot\:(4.601\cdot\:\text{V}\text{B}+23.99)-{\text{e}}^{{\text{c}\text{o}\text{s}}^{2}\left(\text{M}\text{n}\right)}\cdot\:{\text{e}}^{-\text{M}\text{n}}\cdot\:(\text{M}\text{n}+\text{V}\text{M})\cdot\:0.5545\\&\quad-\text{s}\text{i}\text{n}\left(\text{s}\text{i}\text{n}\right(\text{C}\left)\right)\cdot\:{\text{e}}^{-\text{C}}\cdot\:(\text{V}\text{B}+5.42)\cdot\:21.27+0.0005094\cdot\:\text{M}\text{n}\cdot\:\text{V}\text{B}\cdot\:(\text{V}\text{B}\\&\quad+\text{V}\text{T}\text{M})\cdot\:(\text{C}\text{E}+\text{V}\text{M}+\text{c}\text{o}\text{s}(\text{V}\text{M}\left)\right)+32.89\:\:\\\end{aligned}$$

4 $$\begin{aligned}\:\text{U}\text{T}\text{S}&=\:9.421\cdot\:\text{V}\text{M}-84.01\cdot\:\text{C}\text{r}+9.421\cdot\:\text{V}\text{T}\text{M}+1600\cdot\:\text{s}\text{i}\text{n}(\text{s}\text{i}\text{n}(\text{s}\text{i}\text{n}\left(\text{C}\text{E}\right)\left)\right) \\&\quad+{\text{e}}^{-\text{M}\text{n}}\cdot\:\text{t}\text{a}\text{n}\left(\text{C}\right)\cdot\:(\text{V}\text{F}-\text{C}\text{r}+{\text{e}}^{{\text{S}\text{i}}^{2}})\cdot\:185.7\\ &\quad+\text{t}\text{a}\text{n}\text{h}\left(\text{C}\right)\cdot\:\text{t}\text{a}\text{n}\text{h}\left(\text{M}\text{n}\right)\cdot\:({\text{e}}^{-\text{V}\text{T}\text{M}}-\text{C}\cdot\:\text{V}\text{T}\text{M})\cdot\:271.3-289.0\end{aligned}$$

5 $$\begin{aligned}\:\text{T}\text{E}&=56.63\cdot\:\text{C}\cdot\:(\text{M}\text{n}+\text{S}\text{i}-2.663)-378.9\cdot\:\text{s}\text{i}\text{n}(\text{c}\text{o}\text{s}\text{h}(\text{C}\text{r}\left)\right)+\text{t}\text{a}\text{n}\text{h}(\text{C}\text{E}-\text{V}\text{F})\cdot\:\text{c}\text{o}\text{s}\left(\text{C}\text{r}\right)\cdot\:(\text{C}\text{E}\\&\quad-\text{C}\text{r})\cdot\:81.12-(\text{M}\text{n}+\text{V}\text{B})\cdot\:(\text{V}\text{F}-\text{V}\text{M})\cdot\:(\text{C}\text{r}-0.5608)\cdot\:0.01337+368.8\end{aligned}$$

### Interpretation of the trained ML models with SHAP analyses

To better understand the relationship between chemical composition contents (C, CE, Mn, Si, and Cr) and microstructural features (VF, VB, VM, and VTM) with mechanical properties (HER, UTS, and TE), SHAP analysis was employed. This method helps visualize the importance and impact of each input feature on the model’s predictions.

**Fig. 4:**
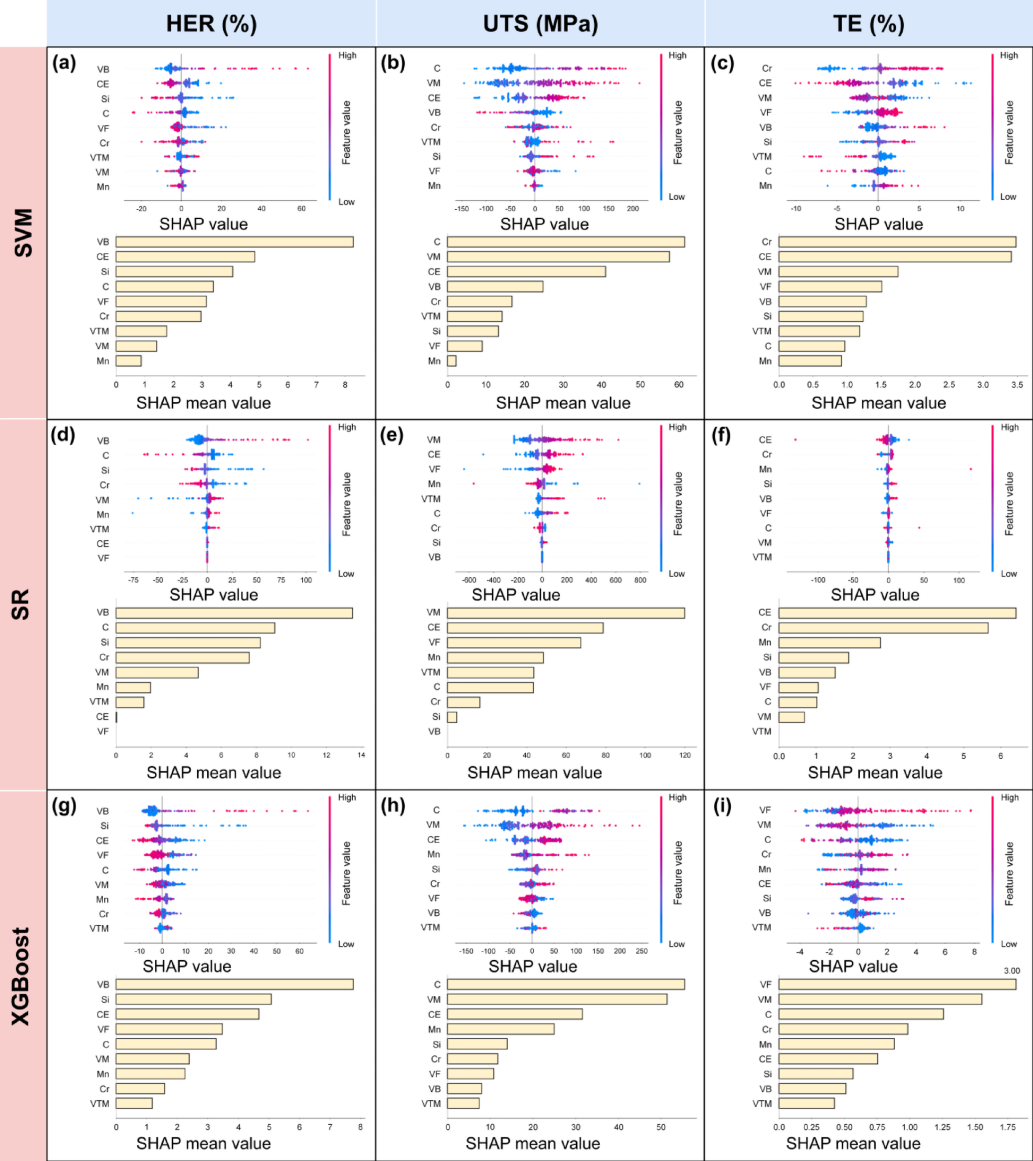
SHAP analysis of the importance of compositions and microstructural features for predicting mechanical properties of AHSS using various ML approaches. (**a-c)** Feature importance derived from SVM models: (**a)** HER, (**b)** UTS, and (**c)** TE. (**d-f)** Feature importance derived from SR models: (**d)** HER, (**e)** UTS, and (**f)** TE. (**g-i)** Feature importance derived from XGBoost models: (**g)** HER, (**h)** UTS, and (**i)** TE. Note: For the SHAP scatter plots, the SHAP value measures the contribution value of a certain feature value for the outcome property, while a red scatter indicates a relatively large feature value, while blue scatter indicates a relatively small feature value. For the SHAP bar plots, the features lying on the upper positions, having larger SHAP mean values, are more influential for the outcome property.

Figure 4 presents SHAP plots for each property across different ML models. The upper scatter plots used color coding (red for high, purple for mean, and blue for low values) to represent feature values, while the position on the x-axis indicates the SHAP value. The lower bar charts display the average SHAP values, indicating overall feature importance.

For HER (Fig. 4a, d and g), all ML models (SVM, SR and XGBoost) identified VB as the most crucial feature. The SVM model, which performed best in regression and prediction tasks, ranks VB as the most important feature, followed by CE. The scatter plot distributions suggest a positive correlation between VB and HER, and a negative correlation between CE and HER. Regarding UTS (Fig. 4b, e and h), SVM and XGBoost models consistently highlight C as the most significant feature, and all ML models indicate that VM also plays a significant role. Given XGBoost’s superior performance in UTS prediction, its emphasis on C and VM appears reliable. The scatter plots for C and VM in SVM, SR and XGBoost models, indicate a positive relationship between C and VM with UTS. In terms of TE (Fig. 4c, f and i), both SVM and SR models consistently identified Cr and CE as the leading important features. The scatter plot distributions across all these three models (SVM, SR and XGBoost) suggest a positive correlation between Cr and TE, and a negative correlation between CE and TE, with these relationships being particularly pronounced in SVM and SR models. Considering SVM is the best predictive model for TE, its indication of Cr as the most important feature is reliable.

### Analysis of MOO solutions with ML models

Multi-objective optimization was employed to determine the optimal combinations of compositions and microstructural features (C, CE, Mn, Si, Cr, VF, VB, VM, and VTM) for achieving the superior mechanical property combination of HER, UTS and TE. Using the best ML models (i.e., SVM for HER and TE, as well as XGBoost for UTS) for predicting these properties, Pareto optimal solution points could be determined through the R-NSGA-III algorithm.

**Fig. 5:**
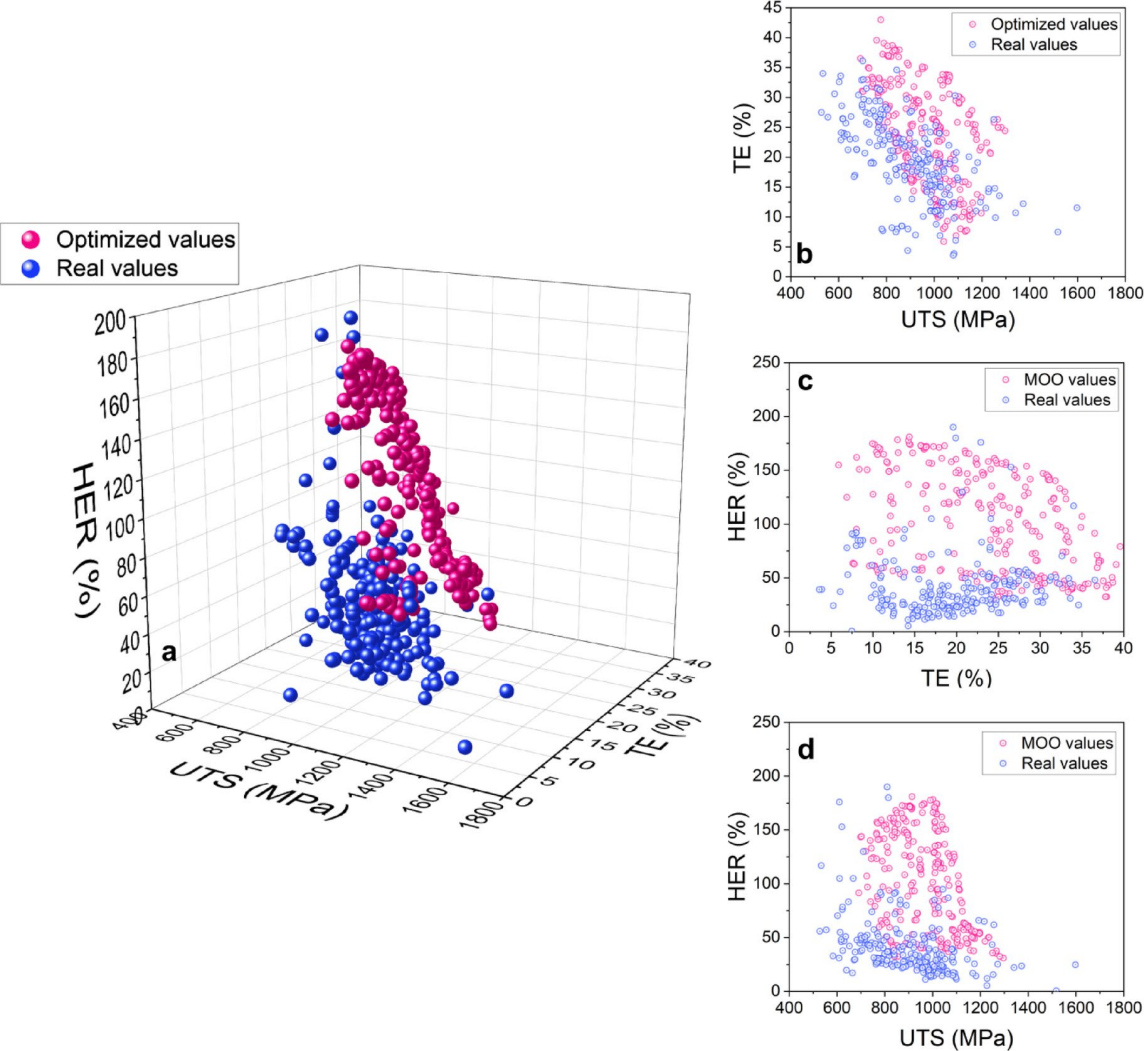
Visualization of optimized and real mechanical property results for AHSS. **(a)** 3D scatter plot of optimized values versus real data regarding HER, UTS and TE of AHSS. (**b-d)** 2D projections of the 3D plot, showing detailed insights in specific property correlations: **(b)** TE and UTS, **(c)** HER and TE, and **(d)** HER and UTS.

Figure 5 illustrates the optimization results. The optimal points show ranges of 31.0–181.0% for HER, 691.3–1296.1 MPa for UTS, and 5.9–43.0% for TE. Figure 5a reveals conflicting relationships between HER, UTS, and TE in the distribution of optimal solutions. The Pareto Front points in Fig. 5b and c, and 5d demonstrate negative correlations of TE-UTS, HER-TE, and HER-UTS, respectively. The strength of these negative associations varies, with HER and UTS showing the strongest inverse relationship, while HER and TE exhibit the weakest. Notably, the optimal solution points generally outperform the original dataset in terms of HER, UTS, and TE, indicating improved performance over existing steel conditions.

**Fig. 6:**
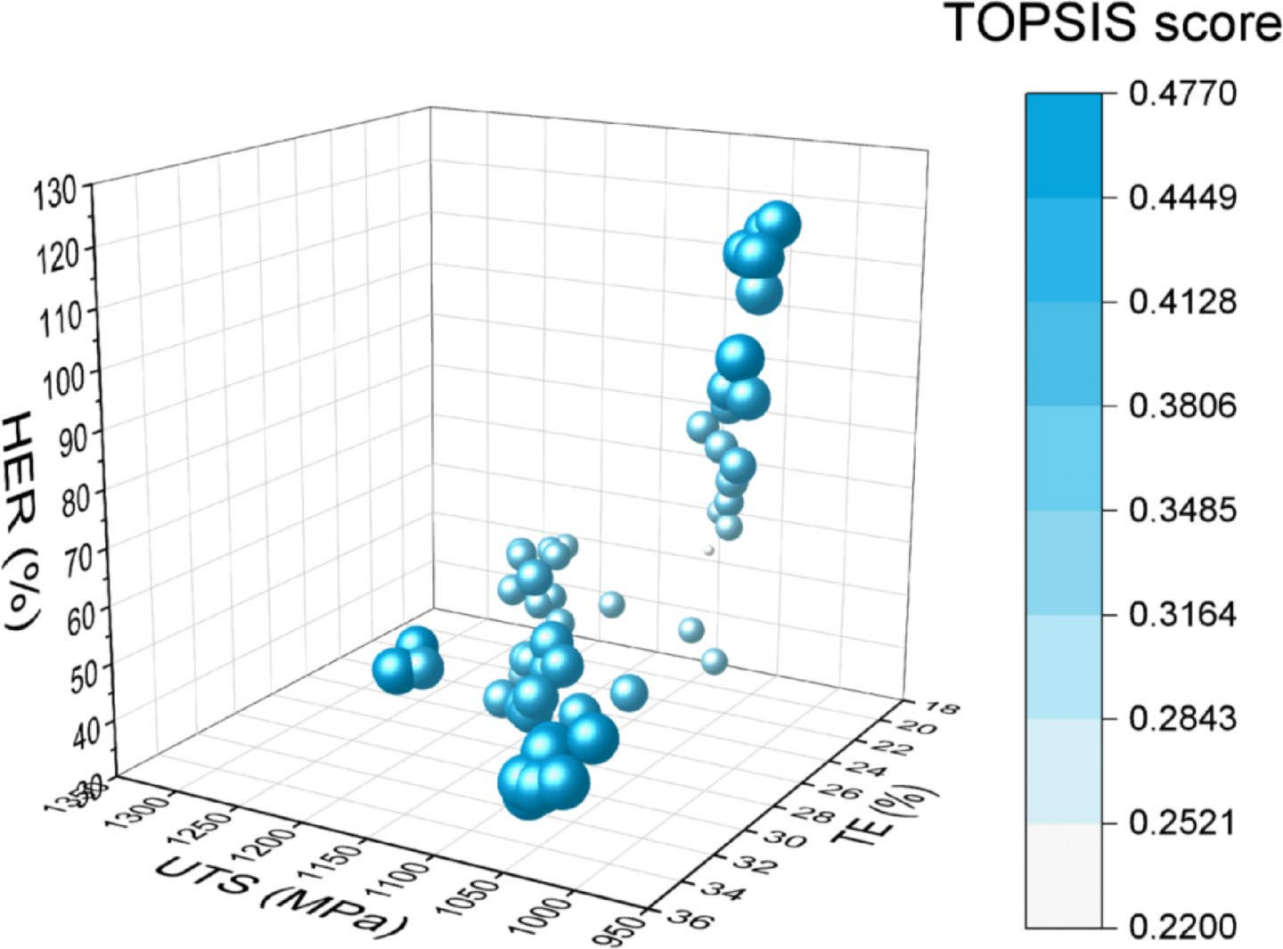
3D scatter plot showing key mechanical properties (HER, UTS and TE) and corresponding TOPSIS scores of industrially experienced selected (IES) steel conditions. Each sphere represents a unique steel condition positioned according to its optimized HER, UTS, and TE values, providing a comprehensive view of the property space occupied by these materials. A blue colormap is employed to represent the TOPSIS score of optimized steel condition. Darker blue indicates higher TOPSIS scores, representing better overall performance across the three properties. Also, the size of each data point is square proportional to its corresponding TOPSIS score, offering an additional visual cue for the overall performance of each condition.

To identify more practical steel conditions, selection criteria based on industrial experience were applied. The applied criteria included (i) the product of UTS and TE exceeding 22,000 MPa × 100% and (ii) UTS over 980 MPa. As illustrated in Fig. 6, these selections show ranges of 31.0–119.8% for HER, 992.6–1296.1 MPa for UTS, and 20.5–33.8% for TE, with average values of 60.4%, 1096.1 MPa, and 26.8%, respectively. The product of UTS and TE ranges from 22,168.5 to 35,852.8 MPa × %, averaging 29,224.5 MPa × %. The compositions and microstructural features range are listed as follows: C (0.11–0.16 wt%), CE (0.31–0.64 wt%), Mn (0.31–2.43 wt%), Si (0.10–1.61 wt%), Cr (0.42–0.95 wt%), VF (0.3–34.5%), VB (0–70.9%), VM (23.3–97.1%), and VTM (0.1–38.5%), with average values of 0.13 wt%, 0.45 wt%, 0.98 wt%, 0.79 wt%, 0.55 wt%, 14.2%, 17.4%, 59.3%, and 9.0%, respectively.

These selections were further analyzed employing an entropy-weighted technique for order preference by similarity to ideal solution (TOPSIS) ranking method. This approach allowed for evaluating the performance of various steel conditions based on the combination of HER, UTS and TE. Figure 6 illustrates the distributions of the properties for industrially experienced selected (IES) steel conditions, along with the corresponding TOPSIS scores. Higher TOPSIS scores indicate better comprehensive mechanical behaviors. The visualization of the TOPSIS analysis results exhibit a triangular distribution of IES points, the closer to the corners, the larger the point sizes, with this effect being particular obvious on the corner of higher TE values. Therefore, when specific IES steel conditions have larger values in either one of HER, UTS or TE, especically TE, the TOPSIS values of these steel conditions are also larger. Steel conditions with the highest TE values generally achieved the highest TOPSIS scores, indicating superior overall performance. Among the studied steel conditions, those with HER values around 40%, UTS around 1050 MPa, and TE around 33% emerged as the top performers. Considering our strong demand on HER, we select the steel conditions with the highest HER values and a bit lower TOPSIS values as the highest-performing steel conditions. Table 2 presents the TOPSIS scores and rankings for the five highest-performing steel conditions identified through the optimization process. The top five highest-ranking steel conditions with respect to HER values exhibited compositions, including 0.12–0.13 wt.% C, 0.40–0.44 wt.% CE, 0.69–1.17 wt.% Mn, 0.10–0.21 wt.% Si, 0.42–0.47 wt.% Cr, with microstructures consisting of 2.9–12.9% ferrite, 52.7–70.9% bainite, 24.2–32.0% martensite, and 0.1–8.3% tempered martensite. These compositional and microstructural combinations resulted in exceptional mechanical properties, with HER ranging from 110.1% to 119.8%, UTS from 1009.5 MPa to 1032.8 MPa, and TE between 22.6% and 24.2%.

## Discussion

### Data imputation and quality

This study started with data preparation, particularly focusing on handling missing values. The choice of missing value imputation method is crucial, as it must maximize information extraction from existing data while remaining practical and rational. Specifically, the imputed values for C, CE, Mn, Si, Cr, VF, VB, VM, and VTM must be non-negative, and the inter-relationships between variables must be preserved. Under this circumstance, MICE emerged as the preferred method due to its iterative optimization process and regularization capabilities. To assess the imputation quality of compositions and microstructural features, a 10-fold random cross-validation was conducted on all non-missing values of C, CE, Mn, Si, Cr, VF, VB, VM, and VTM in the original datasets, which allowed for comparing imputed values with actual hidden values.

**Table 2:** Mechanical properties (HER, UTS and TE) with compositions (C, CE, Mn, Si, and Cr) and microstructure features (VF, VB, VM and VTM) of top-optimized steel conditions using TOPSIS analysis.

Rank	TOPSIS Score	HER (%)	UTS (MPa)	TE (wt%)	C (wt%)	CE (wt%)	Mn (wt%)	Si (wt%)	Cr (wt%)	VF (%)	VB (%)	VM (%)	VTM (%)
1	0.4501	119.8	1013.5	22.7	0.12	0.44	1.10	0.15	0.47	12.1	53.2	26.4	8.3
2	0.4486	118.3	1021.5	22.6	0.13	0.41	1.17	0.13	0.42	3.3	70.8	24.2	1.6
3	0.4600	115.7	1010.8	24.2	0.12	0.42	0.74	0.15	0.47	9.7	61.8	26.7	1.8
4	0.4495	114.8	1032.8	22.9	0.12	0.40	0.69	0.10	0.45	2.9	70.9	26.2	0.1
5	0.4433	110.1	1009.5	24.2	0.12	0.44	1.17	0.21	0.46	12.9	52.7	32.0	2.4

As shown in Fig. 2, the Ridge regression method in MICE achieved an average Q^2^ of 0.9560 for imputing C, CE, Mn, Si, Cr, VF, VB, VM, and VTM. This result compares favorably with previous studies. For example, Li et al.^30^ predicted HER values of AHSS utilizing tensile properties, where the generative adversarial imputation networks (GAIN) method achieved a Q^2^ of 0.85. However, it’s important to note that the model in this work showed some limitations in accounting for ± 20% ratio deviations when the magnitudes of imputed features, particularly VM and VTM, were small. This observation highlights the need for careful interpretation of imputed values, especially when dealing with low phase percentages of minor phases in the microstructure.

### ML models and property prediction

Three ML methods, such as SVM, SR and XGboost, were employed to predict HER, UTS and TE based on the C, CE, Mn, Si, Cr, VF, VB, VM, and VTM. Compared with another two ML models, the SR model provides mathematical expressions of mechanical properties dependent on microstructure characteristics, which allow for further analysis and practical estimations in industrial applications.

To assess the fitting and predictive capabilities of these models, two statistical metrics R^2^ and Q^2^ were employed. For the SR model, a 10-fold cross-validation was applied to optimize parameters, using the highest Q^2^ for function selection. The final models were then developed using the entire datasets, with corresponding R^2^ calculated. As illustrated in Fig. 5, the best performing models were selected based on their R^2^ and Q^2^ values, with Q^2^ given more weight due to its importance in estimating unknown data. Regarding HER, SVM performed best with R^2^ = 0.9518 and Q^2^ = 0.8778. For UTS, XGBoost showed the highest accuracy with R^2^ = 0.9987 and Q^2^ = 0.8913. While, in terms of TE, SVM overperformed ML approaches with the lower overall accuracy (R^2^ = 0.8234, Q^2^ = 0.7181).

These ML models demonstrate significant achievements, there are still discrepancies between predictions and actual values, particularly for TE. These errors can be attributed to variations in experimental settings, sample dimensions, and material conditions. Additionally, the relatively small number of independent features and inherent limitations of ML models contribute to these discrepancies. The prediction errors in regression models may be partially attributed to variations in the processing parameters of different AHSS. Significant differences in the distribution, sizes and morphologies of phases may be observed between two steel conditions with similar phase volume fractions. Despite these challenges, comparable performance metrics have been achieved by the best ML models for HER, UTS and TE. With R^2^ values exceeding 0.6 and Q^2^ values generally above 0.5, it can be concluded that these models are effective and not overfitting. Although exceptionally high predictive accuracy for HER, UTS, and TE (particularly TE) may not have been achieved, reasonably reliable estimates of these properties are provided by prediction models.

Although the SR models didn’t outperform other ML techniques, they provide explicit expressions that offer valuable insights into the relationships between compositional and microstructural features with mechanical properties microstructural features and mechanical properties. The SR-derived equations reveal that the interaction between compositions, phase fractions and HER is complex and nonlinear, rather than being determined by a single factor or following simple monotonic relationships.

### Feature importance and structure-property relationships

SHAP was employed to evaluate the influence and importance of each input variable in predicting HER, UTS and TE, as shown in Fig. 4. This analysis helps uncover the relationships between these properties and bainite volume fraction, carbon content (C), and chromium content, providing insights beyond the complex models of SVM and XGBoost, as well as the intricate mathematical expressions of SR.

The SHAP analysis revealed different patterns for each mechanical property. For HER, VB is considered as the most significant promoting factor, in agreement with previous studies^63^ which suggested that increased VB improved hole expansion performances of multi-phase steels. In contrast, CE and VM were identified as critical depressing factors, consistent with findings^64^ that higher VM of DP steels can induce cracks and lower HER values due to the difference in deformation capacity between soft ferrite and hard martensite. Regarding UTS, C and VM were found to be the leading promoting factor, corroborating earlier research^64^ that higher VM led to higher UTS of DP steels. Interestingly, VB appeared as a significant depressing factor for UTS, which, while seemingly contradictory to some studies on ferrite-bainite steels^65^, may be explained by the complex interactions in multi-phase steels. For TE, Cr was identified as the most significant promoting factor, while CE emerged as the most significant depressing factor.

### Multi-objective optimization and practical implications

The MOO of HER, UTS and TE using the R-NSGA-III algorithm offered opportunity for enhancing comprehensive mechanical behavior of AHSS. The selection of aspiration points based on initial R-NSGA-III results proved crucial in generating superior optimal solutions. Further refinement through domain knowledge and entropy-weighted TOPSIS analysis enhanced the practicality and comprehensiveness of the final solutions. Figure 5a demonstrates the effectiveness of the optimization approach, with optimized solutions (red points) showing consistently higher values for HER, UTS, and TE compared to the original data. However, the distributions in Figs. 5b-d reveal important trade-offs among these properties. The negative relationships observed, particularly strong between HER and UTS, moderate between TE and UTS, and weak between HER and TE, underscore the challenges in simultaneously maximizing all three properties. The inverse relationship between HER and UTS can be attributed to the reduced energy absorption capacity of steels with higher UTS, leading to lower HER values^66^. This trade-off is a critical consideration in AHSS design.

Guided by industrially experienced selected (IES) steel conditions (UTS × TE ≥ 22,000 MPa × % and UTS ≥ 980 MPa), 60 optimized steel conditions (Fig. 6) were identified, which met these criteria. These steel conditions generally exhibited lower HER values compared to the broader set of optimized points, consistent with the observed property trade-offs. Notably, most of these steel conditions featured low bainite (VB) content and high carbon equivalent (CE) content, aligning with the SHAP analysis results shown in Fig. 4b, e and h.

Further refinement using entropy-weighted TOPSIS allowed for identifying the five representative optimal steel conditions, as shown in Table 2. These conditions exhibited a trade-off relationship between values of HER and TE. Specifically, the optimzied steel conditions have the highest HER from 110.1 to 119.8%, and much lower UTS from 1009.5 MPa to 1032.8 MPa, as well as much lower TE from 22.6 to 24.2%, respectively. These steel conditions have much lower concentration of Si and relatively lower Cr. Their major phase is bainite, followed by martensite and ferrite. These phase percentages largely conform to the composition- microstructure-property relationship revealed by SHAP analysis.

## Method

### Data collection

In this work, 212 steel conditions were investigated, forming 212 datasets which are listed in Table S1. These datasets were obtained from earlier research. Each dataset contained 29 features, including compositions, microstructure characteristics and mechanical properties. The compositions include Fe, Nb, Ni, P, N, B, S, V, Ti, Cu, Mo, Al, C, CE, Mn, Si and Cr. The microstructural features consisted of d_F_, VF, VB, VM, and VTM. Concerning mechanical properties, HER, yield strength (YS), UTS, uniform elongation (UE), post uniform elongation (PUE), and TE were collected. Among the 212 datasets, only 28 were complete datasets, while others had varying degrees of missing data. The values of HER, UTS and TE were provided in each dataset. The presence of missing data in the majority of datasets highlights the significance of employing missing data analysis and imputation methods to ensure the robustness and reliability of the derived models.

### Missing value imputation

#### Missing value analysis

**Fig. 7:**
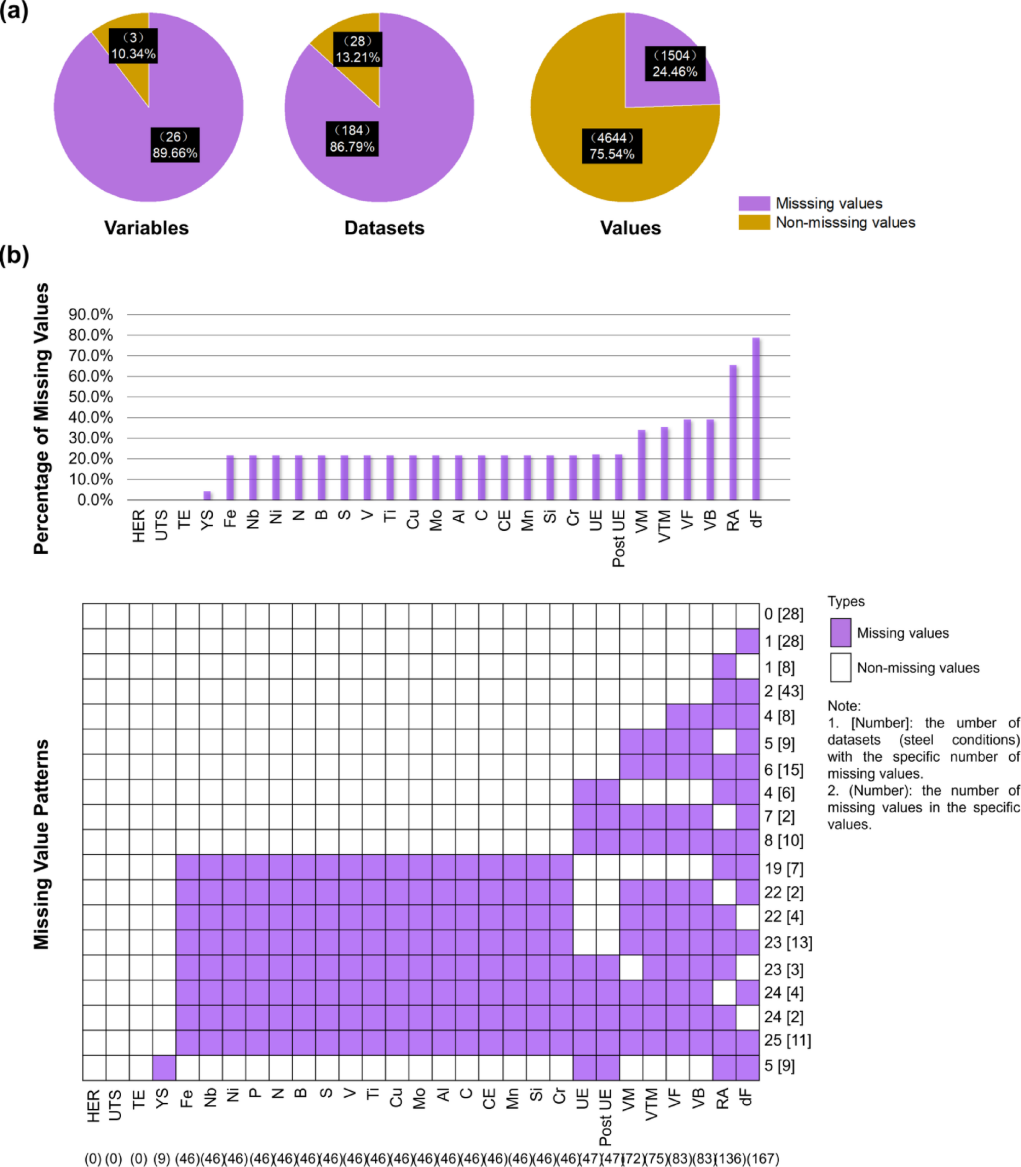
Missing value analysis. (**a)** Percentage of variables, datasets and values with missing values. (**b)** Percentage of missing values for each variable, arranged in ascending order along with patterns of missing data across the dataset, illustrating the distribution and co-occurrence of incomplete information.

The missing value analysis was performed on the acquired experimental data using the missing value analysis function in Statistical Package for the Social Sciences (SPSS) to identify the percentages of variables (features, such as microstructure characteristics and mechanical properties), datasets (steel conditions), and values containing missing values, as seen in Fig. 7a. From Fig. 7a, the missing rates of variables, datasets and values are 89.66%, 86.79% and 24.46%, respectively.

Additionally, the md.pattern() function from the multivariate imputation by chained equations (MICE) package in R was employed to analyze the missing value patterns. Figure 7b show the percentage of missing values in each variable and the missing value patterns. From Fig. 7b, the missing values follow non-monotone patterns, since some missing value blocks are above non-missing value blocks, indicating that missingness of certain variables does not lead to the missingness of all subsequent variables.

The theory of missing value mechanisms was derived from the classification framework developed by Rubin^67^. Missing value mechanisms include missing completely at random (MCAR), missing at random missing (MAR), and missing not at random (MNAR)^68^. Specifically, MCAR indicates that the probability of missingness is unrelated to both observed and unobserved values in the datasets. MAR means that the probability of missingness depends only on observed values, but not on unobserved values. Furthermore, MNAR arises when the probability of missingness is associated with unobserved values. In order to explore the missing value mechanism of the datasets in this study, the expectation maximum (EM) function in the SPSS multiple imputation options was chosen, generating Little’s MCAR chi-square test which helps determine whether the missing value is completely at random. In this work, the significance value of the MCAR test was 0.00, which was lower than 0.05, indicating that the missing data was not completely at random and may be related to other observed or unobserved values^69^ in the 212 datasets. However, distinguishing between MAR and MNAR is not feasible through statistical tests alone, and several suggestions on this case were provide by the literature^70,71^. Cummings^70^ suggested that the assumption of MAR could be reasonable if the datasets are properly sampled. Also, Pedersen et al.^71^ proposed that the assumption of MAR is more plausible when more variables are integrated into the imputation model than the analysis model. Therefore, the missing value mechanism for current 212 datasets can be considered MAR.

#### Multivariate imputation by chained equations (MICE)

**Fig. 8:**
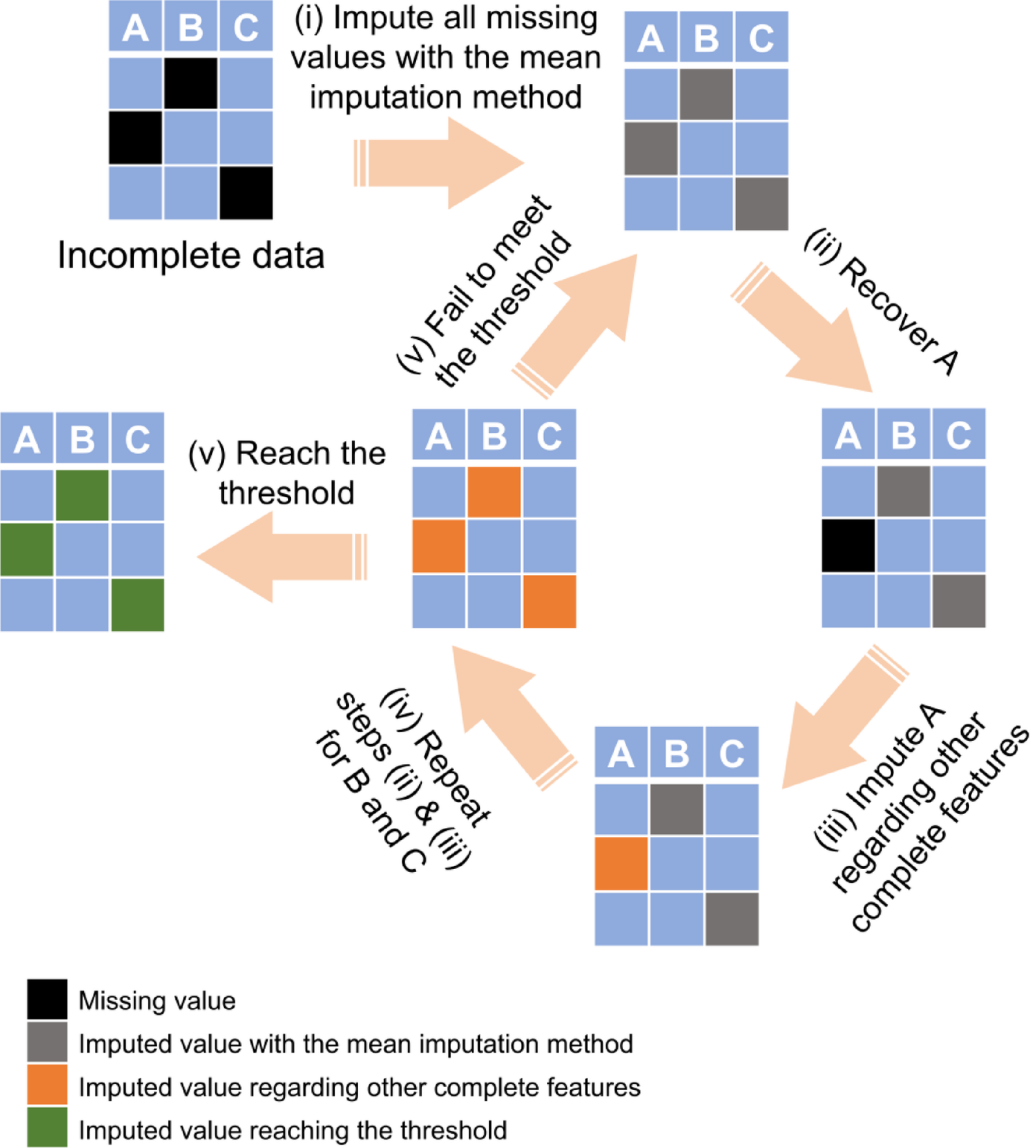
Schematic illustration of MICE algorithm for a dataset with features A, B, and C. The process begins with mean imputation to fill missing values (step i). Next, missing values in feature A are imputed using a regression model (A ~ B + C) to predict and replace them (steps ii and iii). This process is repeated for features B and C (step iv). The algorithm iterates through these steps until a convergence criterion is met, after which the imputation process terminates (step v).

MICE was employed to handle missing data in the datasets consisting of 212 steel conditions. Figure 8represents the schematic illustration of MICE algorithm. In this iterative method, each missing value was initially filled with the mean of its corresponding variable (feature). Then, the imputed values for one variable were temporarily reset to missing. A prediction model was trained using the observed values of this variable as the dependent variable and all other features as independent variables. After that, the missing values of the variable were replaced with estimates from the prediction model. These steps were repeated for all features containing missing values, completing one iteration. The entire process was repeated for multiple iterations to achieve stable imputations^72^.

Three MICE methods were utilized, such as MLR, Lasso, and Ridge regression. The termination criterion for these methods was set as a convergence level where the variation between prior and posterior estimations became smaller than 10^−3^ times the variation of the corresponding variables within the complete (pre-existing) values. Given that microstructural features and mechanical properties are inherently positive, a post-processing step was implemented to convert any negative estimates to zero after each iteration.

Cross-validation tests were conducted to determine the optimal MICE method. Specifically, the observed values in the target features containing missing values were randomly divided into 10 groups. Ten distinct datasets were generated by sequentially replacing one group of observed values with missing values. Each of three MICE methods was applied to these 10 artificially incomplete datasets. The imputed values corresponding to the intentionally hidden observed values were compared with the real values, quantified by Q^2^ metric. To filter the most valuable features for further ML prediction, we tried cross-validation tests on all variables, and select the variables with highest Q^2^, which indicates that these variables are highly associated. As a result, 5 compositions, C, CE, Mn, Si and Cr, and four microstructural features, VF, VB, VM, and VTM, are selected to conduct further MICE cross validation and ML fitting.

Upon selection and application of the best-performing MICE method to the 212 steel datasets, a complete dataset suitable for subsequent regression analysis was obtained.

### Regression

#### Support vector machine (SVM)

**Fig. 9:**
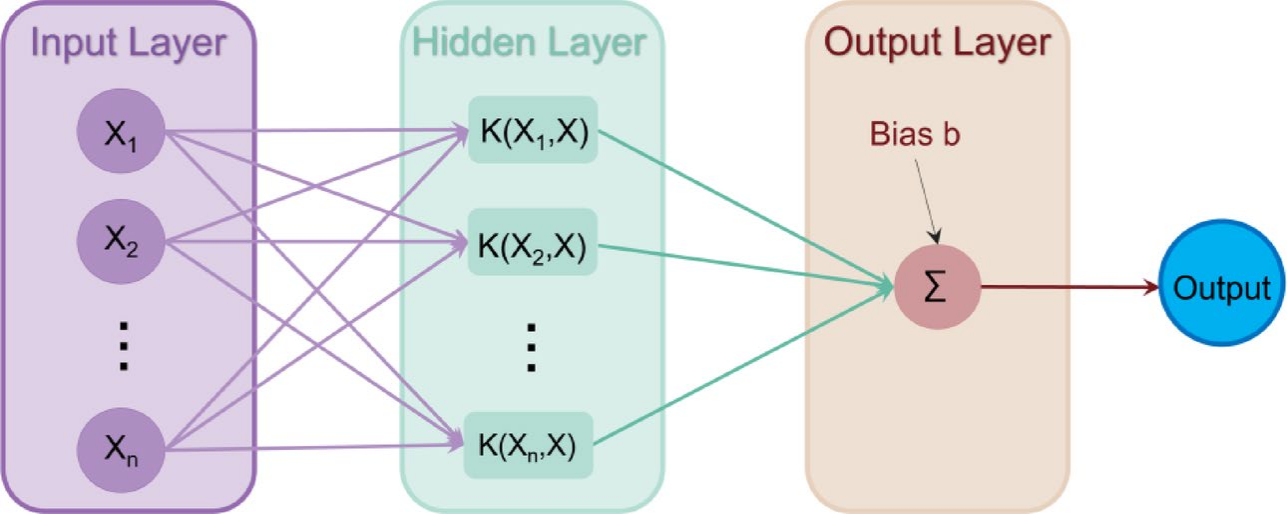
Schematic representation of the SVM structure. The architecture includes an input layer, a hidden layer, and an output layer. The input layer accepts variables X₁, X₂, …, X_n_. In the hidden layer, kernel functions K(X₁, X), K(X₂, X), …, K(X_n_, X) map the input variables to a higher-dimensional space. The output layer combines the kernel results through weighted linear combinations and a bias term (b), producing the final output as the sum of all weighted kernel functions and the bias.

SVM is a powerful ML algorithm used for both classification and regression tasks^73^. Figure 9 shows the schematic representation of the SVM structure. For regression, SVM is known as support vector regression (SVR) and this algorithm aims to find a function f(x) that minimizes $\:\epsilon\:$deviations from actual target values for all training data, while maintaining simplicity^74^. The linear SVR function can be expressed by Eq. (6)^75^,

6 $${\text{f}}\left( {\text{x}} \right)\;{\text{ = }}\;\left.\langle {\text{w ,x}} \right\rangle + b$$

where, w, x and b denote the weight vector, input vector and bias term, respectively. SVM offers advantages over traditional neural networks, particularly in achieving globally optimal results^76^. While SVM may not provide explicit interpretations of underlying mechanisms, it offers a robust approach for the regression task in materials science. The performance of SVR relies heavily on parameter optimization^75^. Key factors include the selection of kernel function type and its parameters, which determine the quality of data projections and regression^75^. Additionally, the regulation parameter C and error magnitude ε play crucial roles in defining the optimal function^75^. The optimization problem for SVR can be formulated as Eq. (7)^75^,

7 $$\begin {aligned} &\text{M}\text{i}\text{n}\text{i}\text{m}\text{i}\text{z}\text{e}\:\frac{\text{1}}{\text{2}}{\parallel\text{w}\parallel}^{\text{2}}\text{+C}\sum\:\left({\xi}_{\text{i}}\text{+}{\xi}_{\text{i}}^{\text{*}}\right)\\&\quad\text{S}\text{u}\text{b}\text{j}\text{e}\text{c}\text{t}\:\text{t}\text{o}:\:{\text{y}}_{\text{i}}\text{-}\left(\text{W}\cdot{\text{x}}_{\text{i}}\text{+b}\right)\leq\epsilon+\xi_{\text{i}}\\&\quad\left(\text{W}\cdot{\text{x}}_{\text{i}}\text{+b}\right)\text{-}{\text{y}}_{\text{i}}\leq\epsilon+\xi_{\text{i}}^{\text{*}}\xi_{\text{i}}\text{,}\\&\quad\xi_{\text{i}}^{\text{*}}\geq\text{0}\end {aligned}$$

where, $\xi_{\text{I}}$ and $\xi_{\text{i}}^{\text{*}}$ are slack variables.

Common kernel functions include linear, polynomial, radial basis function (RBF), and sigmoid, with nonlinear functions often better suited to capture complex interactions in real-world problems^76^. In this study, the RBF was employed as the kernel function for our SVM model. Since it was chosen for its constrained complexity, which enhances the model’s generalization performance^76^. The corresponding SVM regression f and its RBF kernel K is given by Eq. (8)^77^,

8 $${\text{f}}({\text{x}},\;{\text{x}^\prime}){\text{ = }}\sum\limits_{i = 1}^n {{\alpha _i}K\left( {x,\;{x_i}} \right)} {\text{ + b}}\\\text{K}\text{(x,}\text{}{\text{x}}_{\text{i}}\text{)=exp(}\frac{\text{-}{\parallel\text{x-}{\text{x}}_{\text{i}}\parallel}^{\text{2}}}{2{\sigma\:}^{2}})$$

where, $\:{{\upalpha\:}}_{\text{I}}$ are Lagrangian multipliers and $\:{\upalpha\:}\:=\:{\left[{{\upalpha\:}}_{1},\:{{\upalpha\:}}_{2},\:\cdots\:,\:{{\upalpha\:}}_{\text{n}}\right]}^{\text{T}}$. Moreover, this selection is based on a thorough parameter optimization process, ensuring the best fit for the specific problem of predicting stretch-flangeability of AHSS.

#### Symbolic regression (SR)

**Fig. 10:**
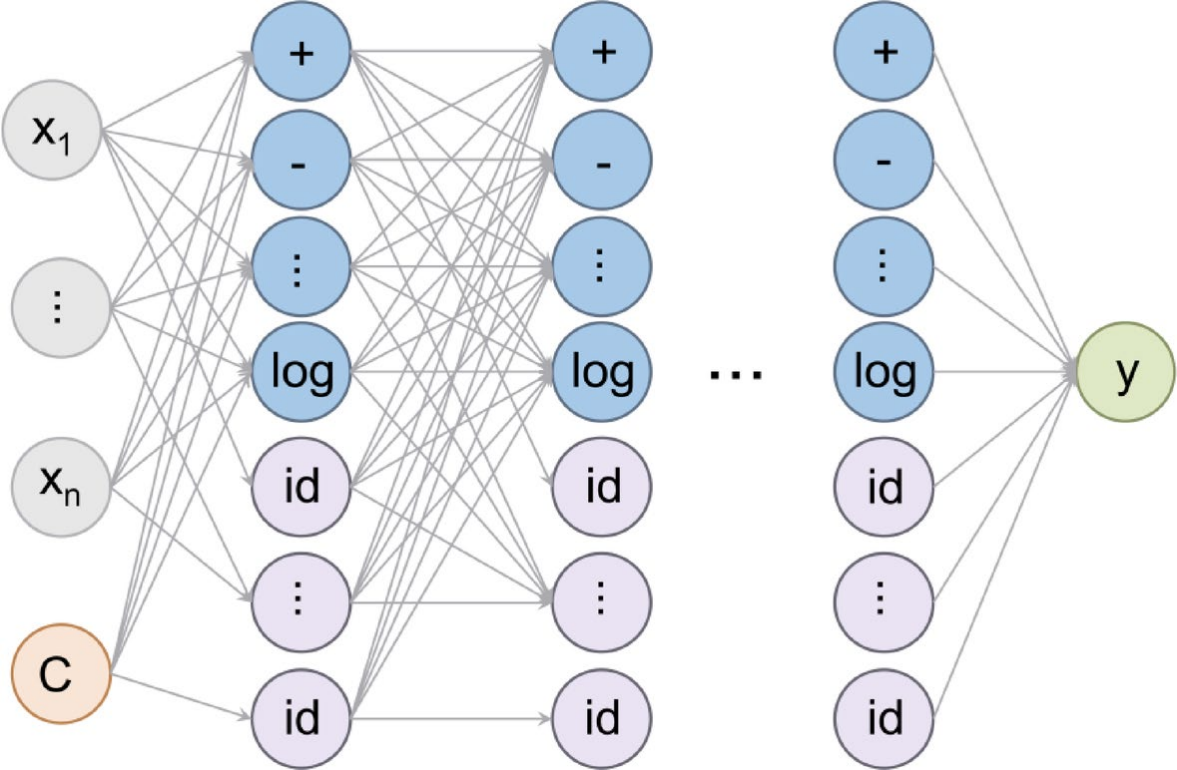
Schematic representation of a Symbolic Regression (SR) architecture with multiple layers. The structure includes an input layer, several hidden layers, and an output layer. The input layer takes features X₁, X₂, …, X_n_, along with a constant term C. The hidden layers perform mathematical operations such as addition, subtraction, logarithms, and identity functions on the input features or outputs from previous layers. The output layer combines the results from the final hidden layer to produce the final prediction.

SR is an advanced ML method that explores explicit mathematical relationships between variables. The schematic illustration of a SR architecture with multiple layers was indicated in Fig. 10. Unlike traditional approaches such as random forest, SR provides researchers with optimal combinations of mathematical operators, variables, and constants to characterize data^78^. The general form of an SR model can be expressed as Eq. (9):

9 $$\text{y=f(}{\text{x}}_{\text{1}}\text{,}{\text{x}}_{\text{2}}\text{,}\cdots\text{,}{\text{x}}_{\text{n}}\text{)}$$

where, y is dependent variable (i.e., HER, UTS and TE), and x_1_, x_2, …,_ x_n_are independent variables (microstructure features). SR is further enhanced through the application of genetic programming (GP), a technique inspired by biological evolution. GP in SR employs strategies of reproduction, mutation, and survival of the fittest to generate and refine populations of mathematical formulas^79^.

In this work, the Genetic Programming Toolbox for the Identification of Physical Systems, version 2 (GPTIPS2), developed on the MATLAB platform, was utilized. GPTIPS2 implements a Multigene Genetic Programming (MGGP) algorithm, which offers significant advantages over simple SR in terms of flexibility and predictive performance^80^. The MGGP model can be expressed as Eq. (10)^80^,

10 $$\text{y}={\text{a}}_{\text{0}}\text{+}{\text{a}}_{\text{1}}{\text{g}}_{\text{1}}\left(\text{x}\right)\text{+}{\text{a}}_{\text{2}}{\text{g}}_{\text{2}}\left(\text{x}\right)+\cdots+{\text{a}}_{\text{n}}{\text{g}}_{\text{n}}\left(\text{x}\right)$$

where, a_0_ represents a bias term, a_i_ denotes the ith scaling coefficient, and g_i_(x) denotes the ith nonlinear transformation of independent variables (genes). Each gene is an independent SR model, the typically of the form is given by Eq. (11)^80^,

11 $${\text{g}}_{\text{i}}\left(\text{x}\right)\text{=f}\left({\text{x}}_{\text{1}}\text{,}{\text{x}}_{\text{2}}\text{,}\cdots\text{,}{\text{x}}_{\text{k}}\right)$$

where, f is a nonlinear function discovered by the GP process, and k<< n(the number of independent variables). GPTIPS2 can identify relatively important variables when presented with a large number of independent variables, contributing to both predictive accuracy and expression simplicity^80^. The importance of each variable can be quantified using techniques such as frequency of occurrence in the best models or sensitivity analysis. These features make GPTIPS2 particularly suitable for the complex materials science application, where multiple microstructural characteristics may influence stretch-flangeability in non-linear ways.

#### Extreme gradient boosting (XGBoost)

**Fig. 11:**
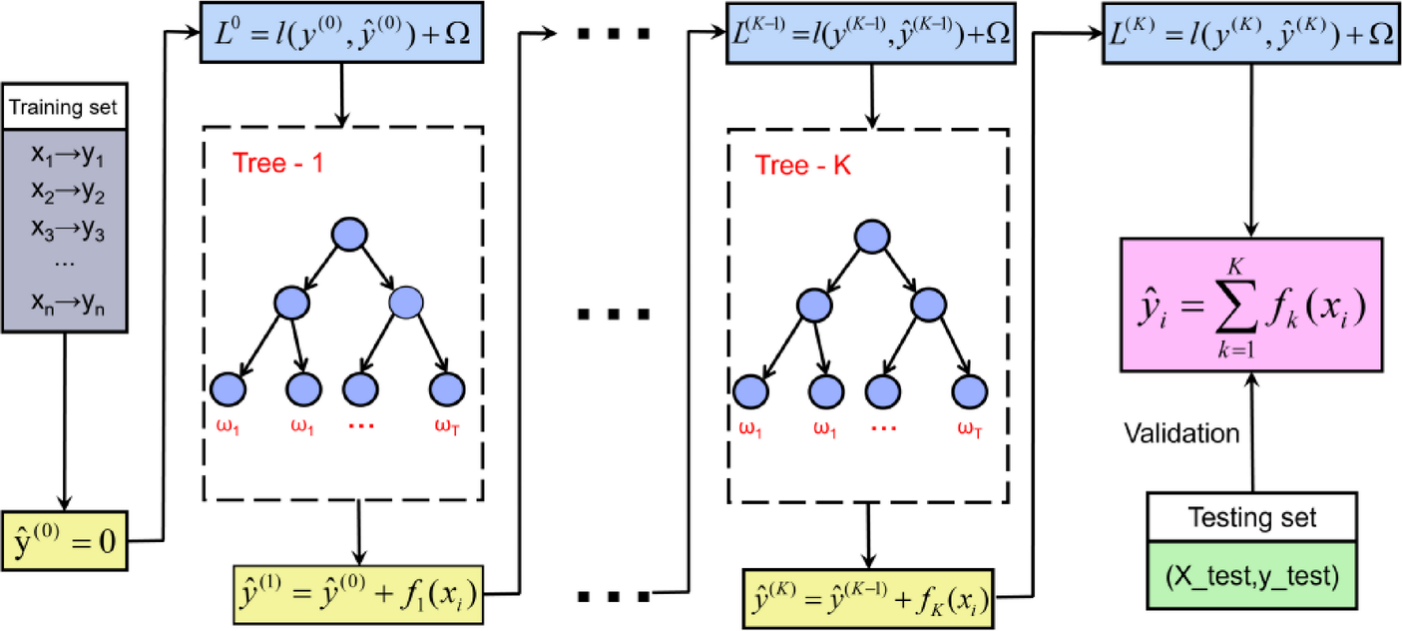
Schematic illustration of the XGBoost algorithm. This process begins with an initial prediction of $\:{\widehat{\text{y}}}^{\left(0\right)}=0$, and sequentially builds decision trees to minimize the loss function $\:\text{L}$, which combines prediction error $\:\text{l}$(from $\:\text{l}({\text{y}}^{\left(0\right)},\:{\widehat{\text{y}}}^{\left(0\right)}),\:\text{l}({\text{y}}^{\left(1\right)},\:{\widehat{\text{y}}}^{\left(1\right)})\:$… to $\:\text{l}({\text{y}}^{\left(\text{K}\right)},\:{\widehat{\text{y}}}^{\left(\text{K}\right)})$) and a regularization term Ω. Each tree $\:{\text{f}}_{\text{k}}\left({\text{x}}_{\text{i}}\right)$ is built to predict the residuals of the previous iteration, with the final output being the summation of contributions from all trees. This iterative process would continue until convergence, with validation of test set ensuring the model’s generalization.

XGBoos is an advanced gradient boosting framework well-known for its efficiency and wide applicability among ML approaches^81^. Figure 11shows the schematic illustration of the XGBoost algorithm. XGBoost is fundamentally based on ensemble learning, especially decision tree ensembles, where the final prediction consists of multiple tree outputs. The algorithm’s core strength stems from its utilization of a second-order Taylor expansion of the loss function^82^. This approach, known as gradient boosting, iteratively refines the model by introducing new trees that address residual errors from previous iterations^82^. The objective function of XGBoost, which the algorithm seeks to minizine, is given by Eq. (12)^81^,

12 $$\text{L}\left(\varnothing\right)\text{=}\sum\:_{\text{i=1}}^{\text{n}}\text{l}\left({\text{y}}_{\text{i}}\text{,}\:{\widehat{{\text{y}}_{\text{i}}}}^{\text{(t-1)}}\text{+}{\text{f}}_{\text{t}}\text{(}{\text{x}}_{\text{i}}\text{)}\right)+\Omega {\text{f}}_{\text{t}}\text{)}$$

where, y_i_ represents the ith observed dependent variable, $\:{\widehat{{\text{y}}_{\text{i}}}}^{\text{(t-1)}}$ denotes the ith prediction from the previous iteration, x_i_ is the ith independent variable, l means the loss function quantifying prediction error between y_i_ and $\:{\widehat{{\text{y}}_{\text{i}}}}^{\text{(t-1)}}$, f_t_ is greedy function to minimize the objective, and Ω represents the penalty function for reducing the complexity of the model. Also, the second order Taylor expansion function for $L\varnothing$ is formulated as Eq. (13)^81,82^,

13 $$\text{L}\left(\varnothing\right)\approx\sum\:_{\text{i=1}}^{\text{n}}\left[\text{l}\left({\text{y}}_{\text{i}}\text{,}\:{\widehat{{\text{y}}_{\text{i}}}}^{\text{(t-1)}}\right)\text{+}{\text{g}}_{\text{i}}{\text{f}}_{\text{t}}\left({\text{x}}_{\text{i}}\right)\text{+}\frac{\text{1}}{\text{2}}{\text{h}}_{\text{i}}{\text{f}}_{\text{t}}\left({\text{x}}_{\text{i}}\right)\right]+\Omega{\text{f}}_{\text{t}}\text{)}$$

where, g_i_ and h_i_ are the first and second derivatives of the loss function with respect to $\:{\widehat{{\text{y}}_{\text{i}}}}^{\text{(t-1)}}$, respectively.

XGBoost’s advanced features make it particularly suitable for complex materials science applications. XGBoost’s ability to handle sparse datasets is particularly valuable through the integration of tree nodes’ indication, allowing the algorithm to efficiently process and learn from incomplete datasets often encountered in materials characterization. To enhance model generalization and prevent overfitting, XGBoost implements a penalty on model complexity. Furthermore, XGBoost employs cache-aware strategies to optimize memory usage and reduce computation time^81^. A key strength of XGBoost lies in its ability to approach target values both exactly and approximately^81^. This flexibility enhances its predictive capabilities across various AHSS compositions and processing conditions, capturing both linear and non-linear relationships between microstructural features and stretch-flangeability.

#### Shapley additive explanations (SHAP)

ML models, including SVM, SR, and XGBoost, have excellent capability of extracting effective information from known data and predicting unexplored data. However, SVM and XGBoost have obscure internal structures and low interpretability, and SR models typically produce complex nonlinear mathematical expressions. To address this limitation, SHAP was utilized in this study. SHAP is an effective approach to explain the impact and importance of each input feature and outcome property. Specifically, SHAP employs a linear addition of input features to interpret the attributions of each feature across all data samples in Eq. (14)^83^.

14 $$\:\text{f}\left(x\right)=g\left({x}^{{\prime\:}}\right){\text{=}\varphi}_{\text{0}}\text{+}\sum\:_{\text{i=1}}^{\text{p}}\varphi_{\text{i}}{\text{x}}_{\text{i}}{\prime\:}$$

where, $\:\text{f}\left(\text{x}\right)$indicates the original model, $\:{x}^{{\prime\:}}$ means the binary simplification of input features x, where 1 indicates the feature is used and 0 indicates it is not, and $\:g\left({x}^{{\prime\:}}\right)$ represents the explanation model approaching the output of $\:\text{f}\left(\text{x}\right)$, p is the number of input features, $\:{{\upphi\:}}_{0}$ represents a constant, and $\varphi_{\text{I}}$ indicates the contribution of feature $\:{\text{x}}_{\text{i}}$. The contribution of each feature x_i_ towards the model f(x_i_) is denoted by $\:{{\upphi\:}}_{\text{I}}$, calculated by Eq. (15)^83^,

15 $$\varphi_{\text{i}}\text{(f,x)=}\sum\:_{{\text{z}}^{{\prime\:}}\subseteq\:{x}^{{\prime\:}}}\frac{\left|{\text{z}}^{{\prime\:}}\right|!\left(\text{p}-\left|{\text{z}}^{{\prime\:}}\right|-1\right)!}{\text{p}!}\text{[}{\text{f}}_{\text{x}}\text{(z}{\prime\:}\text{)-}{\text{f}}_{\text{x}}\text{(z}{\prime\:}\text{i)]}$$

where, $f_x(z^{\prime})$ is equal to $\:\text{f}\text{(z)}$, with $\:\text{z}$’ being the binary simplification of z. The resulting $\:{{\upphi\:}}_{\text{I}}$values equivalent to Shapley values in game theory, representing each input feature’s contribution to the outcome property, when multiple features are involved. SHAP assesses the importance of each input feature by altering input values and calculating the sensitivity of predicted outcomes’ errors. Particularly, features that cause larger deviations in prediction errors are considered more important^84,85^.

### Multi-objective optimization (MOO)

A MOO approach was also employed in this study to address the challenge of simultaneously optimizing multiple, often conflicting material properties of AHSS^86^. This approach is particularly relevant in materials science, where improving one property may come at the expense of another. For instance, maximizing both UTS and TE in DP steels often involves trade-offs.

The MOO framework developed in this study incorporated estimates for HER, UTS and TE, Specifically, the ML models with the best predicting performance are selected in MOO, namely, SVM model for HER and TE, and XGBoost model for UTS. The relevant constraints are taken into consideration. These constraints ensured all variables were non-negative, the acceptable ranges for C, CE, Mn, Si, and Cr are 0.05–0.25 wt.%, 0–0.8 wt.%, 0.3–3 wt.%, 0.1–2 wt.% and 0.4–1wt.%, respectively, and that the sum of volume percentages of different phases (ferrite (VF), bainite (VB), martensite (VM), and tempered martensite (VTM)) equaled 100%, with tolerance limit of ± 0.1%. The optimization process aimed to identify a Pareto front, comprising non-dominated solutions while improving one objective necessarily compromised another^87^.

To achieve this, the R-NSGA-III, an advanced evolutionary algorithm designed for MOO, was applied. R-NSGA-III is developed based on NSGA-III, with a distinct reference points generation strategy that based on aspersion that user provides^88^. In this study, the aspersion points (ref_points) were developed based on the 212 real steel conditions collected from literature, with population size per reference point (pop_per_ref_point) of 153, selection scale (mu) of 0.9, and maximum generation of 100 to ensure better MOO performances.

The R-NSGA-III process started with a randomly generated population. Then it calculated the intercepts of the unit hyperplane with the normalized aspiration points. After that, the Das and Dennis points were generated on the unit hyperplane, which were subsequently adjusted to create reference points. The iterative process involves normalizing the population, assigning solutions to reference directions, selecting solutions, performing genetic operations, and generating offspring. This iteration continues until a threshold condition is met, at which point the final optimal points are determined^88^.

### Regression model evaluation metrics

In order to evaluate the performance of the regression models, several critical metrics were employed, such as the total summation of variances (TSS), summation of residual squares (RSS) and summation of squared predictive residuals (PRESS). These metrics were calculated using real values (y_i_), predicted values in the training set ($\:{\widehat{\text{y}}}_{\text{i}}$) and predicted values in the test set $\:{\widehat{\text{y}}}_{\text{i}\text{p}}$, and the mean value ($\:\stackrel{\text{-}}{\text{y}}$) of the dependent variable. The coefficient of determination, R^2^, measures how well the statistical model fits the data, which is given by Eq. (16)^89^,

16 $${\text{R}}^{\text{2}}\text{=1-}\frac{\text{RSS}}{\text{TSS}}\text{=1-}\frac{{\sum}_{\text{i=1}}^{\text{n}}{\text{(}{\text{y}}_{\text{i}}\text{-}{\widehat{\text{y}}}_{\text{i}}\text{)}}^{\text{2}}}{{\sum}_{\text{i=1}}^{\text{n}}{\text{(}{\text{y}}_{\text{i}}\text{-}\stackrel{\text{-}}{\text{y}}\text{)}}^{\text{2}}}\text{}$$

R² represents the proportion of variance in the dependent variable that is predictable from the independent variables, ranging from$-\infty$to 1^89^. Therefore, a higher R² value indicates a better fit of the regression model to the data, with R^2^ larger than 0.6 can be considered ideal^90^. Additionally, Q was employed to assess the model’s predictive performance. Q^2^ is calculated similarly to R² but used the predicted values from the test set. The value of Q² can be calculated by Eq. (17)^91^,

17 $${\text{Q}}^{\text{2}}\text{=1-}\frac{\text{PRESS}}{\text{TSS}}\text{=1-}\frac{{\sum}_{\text{i=1}}^{\text{n}}{\text{(}{\text{y}}_{\text{i}}\text{-}{\widehat{\text{y}}}_{\text{ip}}\text{)}}^{\text{2}}}{{\sum}_{\text{i=1}}^{\text{n}}{\text{(}{\text{y}}_{\text{i}}\text{-}\stackrel{\text{-}}{\text{y}}\text{)}}^{\text{2}}}\text{}$$

Q² is ranging from $-\infty$to 1, and a higher Q² value suggests better predictive capability of the model for new, unseen data^91^. Q² can be interpreted as a cross-validated R² and is particularly useful for assessing the generalizability of models. Q^2^ larger than 0 means that the model has predictive power, and Q^2^ larger than 0.5 can be considered as an indication of high predictive ability^92^. To ensure accurate evaluation of models’ predictive abilities, a 10-fold cross-validation strategy was applied. This approach provides a robust assessment of the model’s performance across different subsets of the data, helping to mitigate overfitting and providing a more reliable estimate of the model’s predictive power^93^.

## Conclusions

In this study, a novel ML framework was established, enabling accurate prediction and optimization of AHSS stretch-flangeability based on composition-microstructure-property correlations. The significant findings of our work are listed as follows.

1. A diverse dataset from 212 steel conditions were collected, ensuring the generalizability of our findings across various AHSS grades. The dataset was carefully analyzed, and missing values were imputed using MICE with Ridge regression, ensuring high imputation quality (average Q^2^ of 0.9560).
2. Advanced ML models were employed in this work, including SVM, SR and XGBoost, to predict key mechanical properties such as HER, UTS and TE. The best-performing models achieved R^2^ values of 0.9518, 0.9987, and 0.8234 and Q^2^ of 0.8778, 0.8913, and 0.7181 for HER, UTS, and TE, respectively, demonstrating their ability to obtain complex composition-microstructure-property relationships.
3. Interpretable insights into the importance of different microstructural features were provided using SHAP analysis. This analysis revealed that bainite volume fraction (VB) is the most significant promoting factor for HER; carbon content (C) and martensite volume fraction (VM) are the most critical factor for UTS; and chromium content (Cr) is the most influential factor for TE.
4. Multi-objective optimization was conducted using the reference point based non-dominated sorting genetic algorithm III (R-NSGA-III), generating 252 optimized steel conditions with improved comprehensive mechanical properties. The optimization results revealed important trade-offs among HER, UTS, and TE, particularly a strong inverse relationship between HER and UTS.
5. Five representative optimal steel conditions were determined using industrially relevant selection criteria, entropy-weighted TOPSIS and objective-wise analysis. These conditions are characterized by a composition of 0.12–0.13 wt.% C, 0.42–0.47 wt.% Cr, 0.69–1.17 wt.% Mn, and 0.10–0.21 wt.% Si, with a carbon equivalent of 0.40–0.44 wt.%. The microstructural features predominantly consist of bainite (52.7–70.9%) and martensite (24.2–32.0%), with smaller proportions of ferrite (2.9–12.9%) and tempered martensite (0.1–8.3%). These optimized conditions demonstrate a desirable combination of mechanical properties, including HER ranging from 110.1% to 119.8%, UTS from 1009.5 MPa to 1032.8 MPa, and TE from 22.6% to 24.2%, providing valuable guidelines for industrial applications.

## Electronic supplementary material

Below is the link to the electronic supplementary material.

Supplementary Material 1

## Data Availability

The data used in this study, consisting of 212 AHSS datasets collected from previously published literature, have been compiled and made available in the Supplementary File. The source data underlying visualizations presented in Figs. 2a-i, 3a-i, 4a-i and 5a-d, and 6, have been deposited in a Figshare repository under https://doi.org/10.6084/m9.figshare.27894048.

## Code availability

The codes utilized in this work are available from the corresponding author upon reasonable request.
